# Chitosan Membranes Filled with Cyclosporine A as Possible Devices for Local Administration of Drugs in the Treatment of Breast Cancer

**DOI:** 10.3390/molecules26071889

**Published:** 2021-03-26

**Authors:** Sonia Trombino, Federica Curcio, Teresa Poerio, Michele Pellegrino, Rossella Russo, Roberta Cassano

**Affiliations:** 1 Department of Pharmacy and Health and Nutrition Sciences, University of Calabria, Arcavacata di Rende, 87036 Cosenza, Italy; sonia.trombino@unical.it (S.T.); federica.curcio@unical.it (F.C.); michele.pellegrino@unical.it (M.P.); rossella.russo@unical.it (R.R.); 2 Institute on Membrane Technology (CNR-ITM), University of Calabria, Arcavacata di Rende, 87036 Cosenza, Italy; t.poerio@itm.cnr.it

**Keywords:** membrane, cyclosporine A, chitosan, carboxylated chitosan, breast cancer

## Abstract

The aim of this work is the design, preparation and characterization of membranes based on cyclosporine A (CsA) and chitosan carboxylate (CC) to be used as an implantable subcutaneous medical device for a prolonged therapeutic effect in the treatment of breast cancer. The choice to use CsA is due to literature data that have demonstrated its possible antitumor activity on different types of neoplastic cells. To this end, CsA was bound to CC through an amidation reaction to obtain a prodrug to be dispersed in a chitosan-based polymeric membrane. The reaction intermediates and the final product were characterized by Fourier transform infrared spectroscopy (FT-IR) and proton nuclear magnetic resonance (^1^H-NMR). Membranes were analyzed by differential scanning calorimetry (DSC) and scanning electron microscopy (SEM). The data obtained showed the effective formation of the amide bond between CsA and CC and the complete dispersion of CsA inside the polymeric membrane. Furthermore, preliminary tests, conducted on MDA-MB-231, a type of breast cancer cell line, have shown a high reduction in the proliferation of cancer cells. These results indicate the possibility of using the obtained membranes as an interesting strategy for the release of cyclosporin-A in breast cancer patients.

## 1. Introduction

Breast cancer is one of the most diffused neoplastic diseases among women, whose therapeutic approach is based on various methods that include surgical removal, chemo- or radiotherapy and treatment with monoclonal antibodies. Innumerable are the side effects deriving from these therapies, and some of them are related to the cytotoxic activity of drugs that act indistinctly both on cancer cells and on healthy ones. The severe side effects, such as nausea, vomiting, diarrhea, mucositis and myelosuppression, decrease the life quality of the patients. One approach to reduce side effects is to localize the drug to tumors and cancer cells. Local administration of anticancer drugs is already clinically applied. [1] In this approach it is beneficial to increase the residence time of a drug at the administration site. This can be achieved through a carrier matrix that remains at the site of administration and releases therapeutic substances in a controlled manner. Examples of systems for local administration are injectable in situ gelling materials [2,3,4], beads [5], inhalable particles [6,7,8], nanocarriers that remain at the site of administration [9,10,11], microneedles [12,13], liquid crystalline phases [14], creams and ointments [15,16] and metal drug releasing implants [17,18]. Nowadays, in the literature, it is reported that the use of subcutaneous implants as drug delivery systems allows to prolong the release of drugs with different characteristics in various parts of the body [19,20,21]. Moreover, to be functional, these devices must show a certain biocompatibility with the reference tissues [22,23]. Natural or synthetic polymeric materials are often used, and possess a high degree of biocompatibility, reduced toxicity, and considerable biodegradability [24,25,26]. For this reason, the aim of this work was to design implantable dense polymeric membranes, able to prolong the release of a drug directly to the site of action, to guarantee a homogeneous distribution of the same and be able to minimize side effects. In this approach, a dense membrane can act as a vector for the prolonged release of a drug when placed in contact with a neoplastic lesion. Dense membranes consist of a dense structure in which the drug can be transported by diffusion under the driving force of a pressure, concentration, or electrical potential gradient. One of the main physical methods to control the release using a polymeric system is membrane permeation controlled (MPC) release. In this system, the drug is incorporated into a polymeric membrane and the rate of drug release depends on its diffusion through the membrane. The drug used in this work was CsA, which is a neutral cyclic polypeptide that is considered a powerful immunosuppressant and used in clinical practice in the prevention and treatment of rejection of organ transplants. For many years it was used as a drug of choice in the topical and systemic treatment of dermatological pathologies (psoriasis, atopic dermatitis, alopecia), of autoimmune ones (lupus), of rheumatoid arthritis and of autoimmune uveitis [27,28]. Recent studies have also demonstrated the possible antitumor activity of this molecule on different types of neoplastic cells [29]. The mechanism of action by which CsA inhibits the proliferation of tumor cells is based on an inhibition of their glycolytic process due to the absence of energy. It exerts a down regulation against the enzyme pyruvate kinase M2, responsible to produce ATP in the final stages of glycolysis [30]. For this reason, as the energy reserves of the cancer cell cease to exist, it is unable to perform the normal functions of the cell cycle, which stops in the G1/S phase, causing cell death. Being a classical immunosuppressant, systemic administration of CsA in patients with breast tumors can compromise the host’s anticancer responses and be harmful. Unfortunately, cyclosporine, a lipophilic drug, is characterized by low solubility and consequent poor oral bioavailability. It also has a narrow therapeutic index and therefore the potential to induce renal toxicity. In this context, an effective, non-toxic, stable and patient-friendly formulation may be useful because it could inhibit the proliferation of breast cancer without affecting general immunity and reducing nephrotoxicity. In particular, the local application of cyclosporine appears to be advantageous as it compromises the suppressive function of the regulatory CD4+, CD25+ and Foxp3 T lymphocytes that infiltrate into tumor tissues [30]. However, this drug, due to its high molecular weight, is unable to distribute evenly and penetrate the stratum corneum of the skin and reach the target site unaffected after its administration. In this regard, the polymer used, chitosan, allows the administration of highly lipophilic drugs, such as CsA, not only orally but also topically. [31]. In particular, the chitosan, a natural polymer derived from chitin, causes an increase in the permeability of the epithelial membrane that favors the permanence of the drug at the absorption site and its paracellular transport [32,33,34]. Specifically, in this work, CsA, after being covalently linked with the carboxylated chitosan, was dispersed inside a polymeric matrix based on chitosan which was subsequently subjected to contact with the target site, facilitating the dispersion of the drug thanks to a high permeability and flow. The reaction intermediates and the final product were characterized by means of Fourier transform infrared spectroscopy and proton nuclear magnetic resonance. The membranes were analyzed by differential scanning calorimetry and scanning electron microscopy. The obtained data showed the effective formation of the amide bond between the CsA and CC and complete dispersion of the CsA inside the polymeric membrane. Preliminary tests conducted on MDA-MB-231, a highly aggressive, invasive and poorly differentiated triple negative breast cancer (TNBC) cell line, have demonstrated antitumor activity considering the high reduction in tumor cell proliferation.

## 2. Results and Discussion

### 2.1. CsACC Synthesis and Characterization

**Figure 1:**
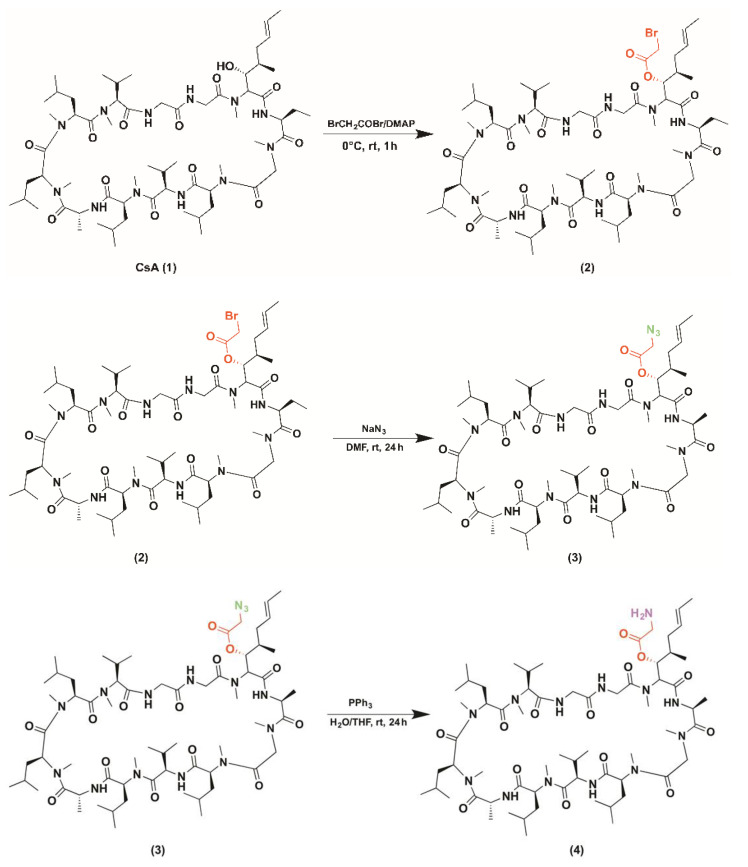

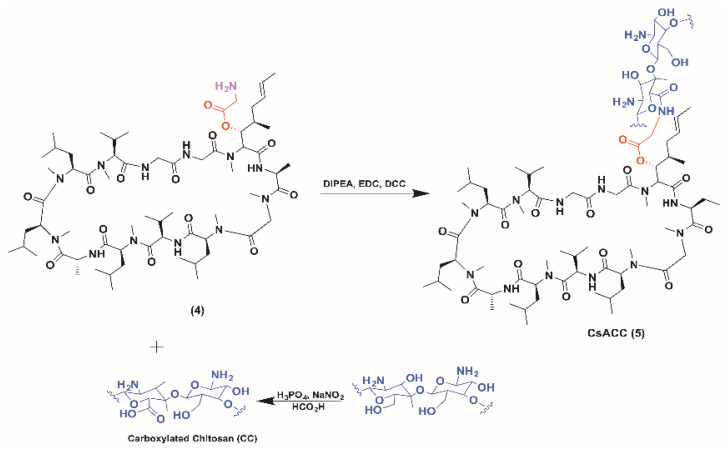
Scheme of CsACC synthesis.

CsACC was obtained through an amidation reaction that favored the covalent bond between the derivatized CsA and the carboxylated chitosan. Cyclosporin A (1) was initially esterified with bromoacetyl-bromide in the presence of DMAP to form the corresponding ester (2). Subsequently, with the addition of sodium azide in the presence of DMF, the bromine atom was replaced by a azide anion (3) to then be reduced to the amine form after addition of triphenyl phosphine, water, and anhydrous tetrahydrofuran (4). The amino group, in the presence of DIPEA, DCC and EDC, showed the formation of a bond with the CC carboxyl group (5) (Figure 1).

**Figure 2:**
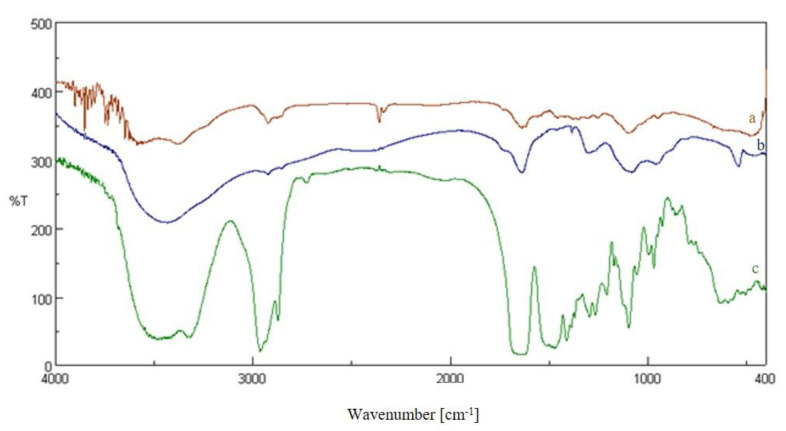
FT-IR of CsACC (**a**), CC (**b**), CsA (**c**).

**Figure 3:**
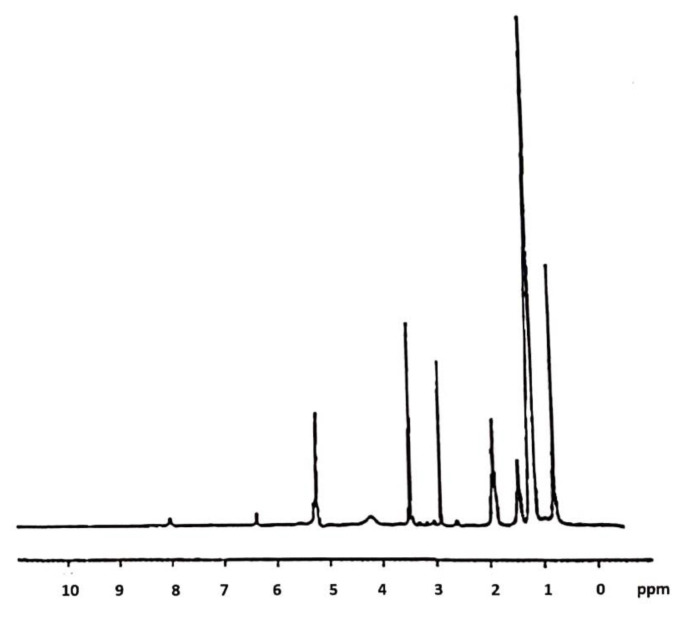
^1^H-NMR spectrum of CsACC.

**Figure 4:**
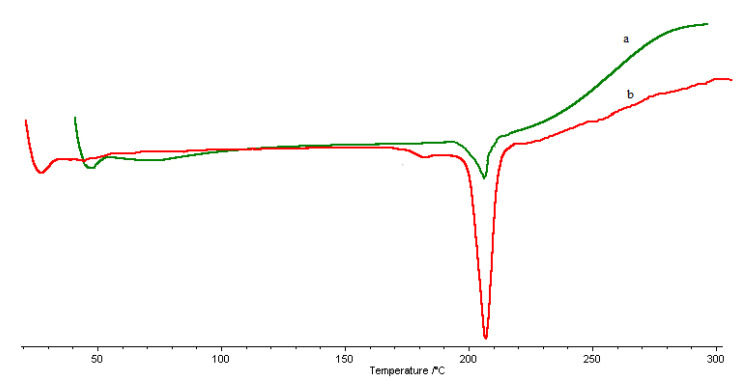
DSC curve of CsACC (**a**), CC (**b**).

The intermediates of the reaction were characterized. The CC was characterized by spectroscopic techniques. FT-IR (KBr) v (cm^−1^): 3435 (OH), 2925, 2855, 2833 (CH aliphatics), 1719(C=O) 1362 (OH) (Figure 2b). ^1^H-NMR (DMSO-d6): 11.2 (bs, 1H), 5.54 (m, 1H), 5.04 (bs, 2H), 4.40 (m, 1H), 3.50 (m, 1H), 3.02 (m, 1H), 2.21 (m, 1H). Yield: 0.8 g. The CsACC derivate was also characterized by FT-IR and ^1^H-NMR. FT-IR spectrum shows the presence of a new band at 1621 cm^−1^ correlated to the stretching vibration of the C=O amide (Figure 2a). ^1^H-NMR analysis has provided a very broad and complex spectrum in CDCl_3_ in which the signals of the *N*-methyl groups (3 ppm) and those related to CH_3_ in the side chain of different amino acid residues (1 ppm) are evident (Figure 3). In this spectrum, the signals of CH_2_ and CH of chitosan between 3–4 ppm are also evident. The DSC curves of the carboxylated chitosan (b) and the CsACC derivative (a) are shown in Figure 4. The CC showed an endothermic peak at 205 °C, the amide derivate at 207 °C, and the CsA (curve not shown) at 244 °C.

**Figure 5:**
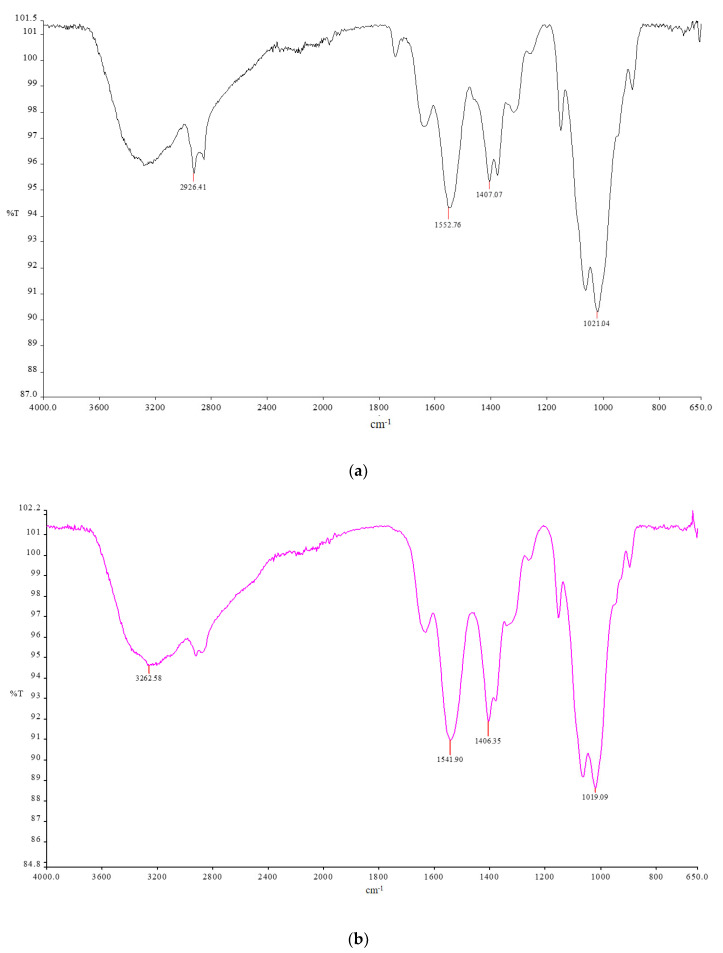
FT-IR of membrane based on chitosan (**a**), chitosan + CsACC (**b**).

The membranes were characterized by FT-IR and electronic scanning microscopy (SEM). Characterization by FT-IR revealed that the spectrum of chitosan did not change, despite the composition of the membranes being modified (Figure 5).

### 2.2. Characterization of Membranes

**Figure 6:**
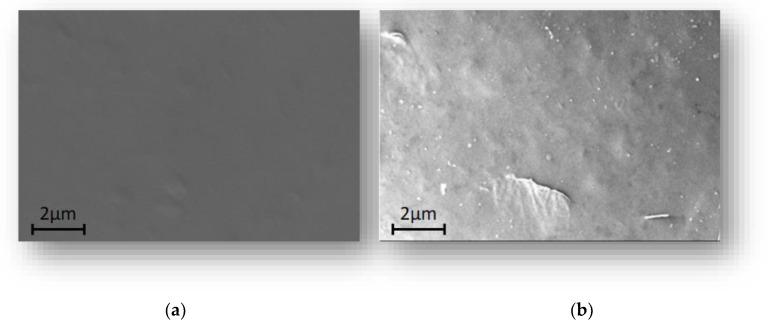
SEM photomicrographies of the membrane based on chitosan (**a**), and chitosan + CsACC (**b**). The images are taken at 10 K× magnification.

The obtained membranes were characterized by SEM micrographies. The results showed that the membrane based on chitosan (a), and chitosan + CsaCC (b) appear dense. However, from figure (b) is evident the presence of the CsACC derivative that appears like filaments that protrude from the membranes (Figure 6).

### 2.3. Skin Permeation Studies

**Figure 7:**
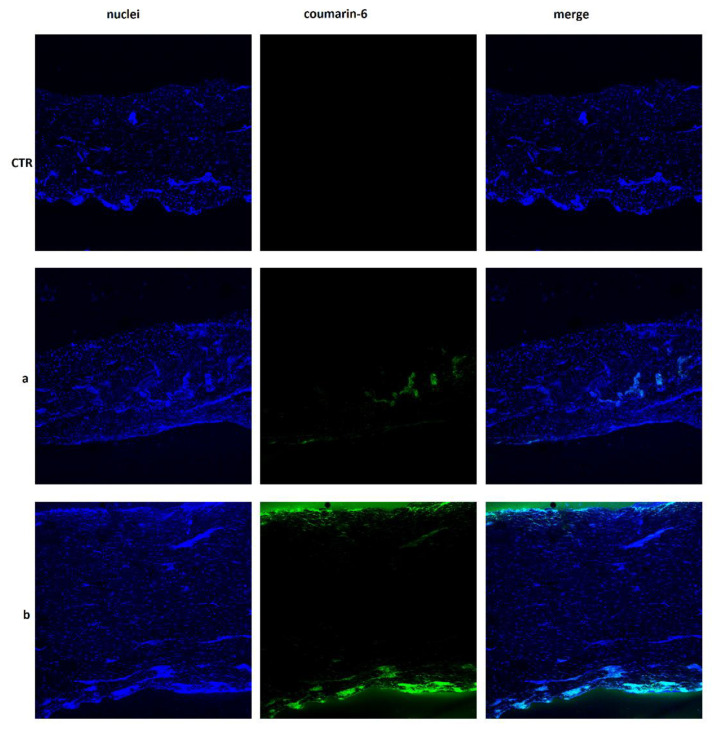
Confocal imagine of chitosan membrane (CTR), membrane based on free CsA containing coumarin-6 (**a**), and membrane based on CsACC containing cumarin-6 (**b**).

To have dermatological formulations and further modulate CsA release, this drug and its prodrug were used to obtain various membranes of chitosan that were then subjected to transdermal release studies to evaluate their potential application and efficacy in oncological diseases treatment. Drug release profiles were evaluated by using Franz diffusion cells with membranes or pigskin. Synthetic membranes, in addition to pig skin, were used because cyclosporine is characterized by a specific absorbance range (λ = 195–215 nm) and, therefore, can interfere with several skin components, such as lipid and proteins, that adsorb at similar wavelengths [35,36]. The presence of skin components in receptor chambers can be attributed to the release medium composition (0.9% NaCl/ethanol 20%) since ethanol can promote phospholipid mobility [37]. Ethanol was added to the release medium since cyclosporine is soluble in ethanol but not in water [38]. To further eliminate the risk of possible interferences, a 14 kDa cut-off membrane was used. In fact, the obtained data showed, by comparing release studies carried out using pig skin and cellulose acetate membrane, an absence of significant interferences. Drug release studies were performed on dermatological formulations at different time intervals (1, 4, 8, 12, and 24 h). Drug release profiles were determined by UV-Vis spectrometry and expressed as percentage of the drug released with respect to the total loaded amount as a function of time. Data showed that, with cellulose acetate membrane, cyclosporin A was released within 8 h from the membranes, in quantities ranging from 0.15% (membrane CHIT + prodrug) to 0.9% (membrane CHIT + CsA) of the total loaded amount. After 1 h, release was not observed from membrane containing free CsA. Instead, the release from membrane containing the prodrug stopped after 4 h. Studies conducted by means of pig skin revealed a total CsA percentage of 4.2% for membrane containing the free CsA and of 2.7% for membrane containing the prodrug, within 24 h. The higher percentage of cyclosporine released using pig skin is probably due to the presence of skin components as previously pointed out. After that, to evaluate if CsA had been released from the membrane in the pig skin, given the low percentage present in the acceptor compartment after carrying out the transdermal release, the chitosan membranes were solubilized and subjected to observation on a UV-Vis spectrophotometer in the range of 195–215 nm. The obtained results revealed the presence of percentage of free CsA and prodrug equal to 21% and 18%, respectively. This result suggests that the remaining portions are placed in the pig skin used during transdermal birth. To validate this hypothesis, chitosan membranes were also prepared using coumarin-6 as a model drug, being a particularly lipophilic substance in analogy to CsA. An observational skin study was performed to verify how coumarin arranged itself within the pig’s skin after transdermal release. Fluorescence microscopy imaging (CLSM) was used to visualize its distribution and penetration depth through the pig skin. As clearly visible in Figure 7, the coumarin-6 was found to be concentrated into the dermis layer of the pig skin after release from membrane containing free CsA as compared to control chitosan membrane. On the other hand, coumarin-6 released from membrane containing the CsA prodrug was positioned both on the shin surface and in the more internal layer, probably the subcutaneous one. Obtained results represent a starting point. Other studies are required to verify with certainty the effectiveness of the prepared membranes and the release mechanisms of CsA.

### 2.4. Cell Proliferation Assays

**Figure 8:**
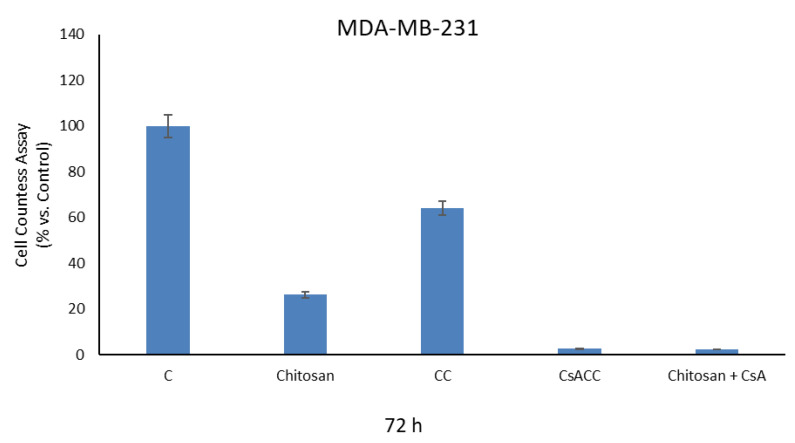
Cell proliferation assay on MDA-MB-231 cell lines of chitosan membranes with and without CsA.

The effects of CsACC membranes and CsA free on the viability processes of cellulose in human breast cancer cells MDA-MB-231, were analyzed by cell proliferation assay. At the concentrations used in the experiment, after 72 h of treatment, significant biological effects were observed in our experimental model. In particular, the results obtained showed a significant decrease in cell viability especially in cells treated with CsACC and with chitosan mixed with free CsA. This effect is not observed by treating the cells uniquely with CsA at the same concentration with which it is present in the membranes (Figure 8).

## 3. Materials and Methods

### 3.1. Materials

For the synthesis of the prodrug, the following materials were used: medium-molecular-weight chitosan purchased from Sigma Aldrich (St. Louis, MO, USA), orthophosphoric acid (H_3_PO_4_) 85% purchased from Carlo Erba Reagenti (Milan, Italy), nitrite of sodium (NaNO_2_), 85% formic acid, bromoacetyl bromide purchased from Sigma Aldrich, sodium bicarbonate (NaHCO_3_), sodium sulfate (Na_2_SO_4_), dimethylaminopyridine (DMAP), dimethylformamide (DMF), phosphate buffer (PBS), sodium azide, diisopropylethylamine (DIPEA), dicyclohexylcarbodiimide (DCC), ethyl 1–3 dimethylaminopropylcarbodiimide (EDC), hydrochloric acid (HCl), sodium chloride (NaCl), and triphenylphosphine purchased from Fluka Chemika-Biochemika (Buchs, Switzerland). The solvents used were: dichloromethane, chloroform, tetrahydrofuran, diethyl ether, ethanol, methanol, acetone and cold ethyl ether, purchased from VWR Chemicals Prolabo (Milano, Italy), Fluka Chemika-Biochemika (Buchs, Switzerland) and LabScan Analytical Sciences (Gliwice, Poland). Methylene blue was purchased from BDH Analytical Chemicals (Ontario, Canada). Ciclosporin (molecular weight 1202.61 g/mol) was purchased from Farmalabor Srl (Bari, Italy). For the preparation of the membranes, the chitosan (average molecular weight) purchased from Aldrich (St. Louis, MO, USA), PVA (Polyvinyl alcohol 13,000–23,000 Dalton, 98% hydrolyzed, Sigma Aldrich), acetic acid purchased from VWR Chemicals Prolabo, multi-well plates purchased from thermo-scientific (Milano, Italy) and distilled water were used.

### 3.2. Cell Culture

Human breast cancer MDA-MB-231 were acquired from Interlab Cell Line Collection (ICLC, Genova, Italy), where they were authenticated. Cells were stored according to supplier’s instructions and used within 6 months after frozen aliquot resuscitations. MDA-MB-231 were cultured in Dulbecco’s Modified Eagle’s medium-F12 plus glutamax containing 5% fetal bovine serum (Invitrogen, Carlsbad, CA, USA), and 1 mg/mL penicillin–streptomycin at 37 °C with 5% CO_2_ air. Mycoplasma negativity was tested monthly (MycoAlert, Lonza, Basel, Switzerland).

### 3.3. Instruments

The 1H-NMR spectra were realized through a Bruker VM 30 spectrophotometer (Bruker, Ettlingen, Germany). The FT-IR spectra were processed through a Jasco 4200 spectrophotometer (Jasco Europe S.R.L, Lecco, Italy). The spectra UV-Vis were recorded with a Jasco V-530 UV/Vis spectrophotometer (Thermo Fisher Scientific, Monza, Italy). Differential scanning calorimetry (DSC) was performed with a DSC 200 PC Netzsch instrument (NETZSCH-Gerätebau GmbH, Verona, Italy). The micrographs of the membranes were carried out using an electronic scanning microscope (SEM) (FELMI-ZFE, Graz, Austria) FEI Quanta 200, FEG (field emission gun) with a 0.5–30 Kv voltage and an EDX Oxford Inca 300 system. The samples were sputtered with gold before the SEM analysis. The solvents were removed using a Buchi Rotavapor R II (Buchi, Cornaredo, Italy), while the lyophilization of some compounds was carried out through an Edwards “Freezing-drying” Micro Modulyo (Thermo Electron Corporation, Gormley, ON, Canada). The evaporation of the membranes and their complete drying were carried out with a stove for vacuum drying (Thermo Fisher Scientific; Waltham; MA; USA).

### 3.4. Synthesis of Carboxylated Chitosan (CC)

In a three-necked flask fitted with a reflux condenser, magnetic stirrer, meticulously flamed under a nitrogen stream (inert atmosphere), 1 g of chitosan and 40 mL of H_3_PO_4_ at 85% were added. After 1 h, 3 g of sodium nitrite (0.043 mol) were added, and the solution was kept under vigorous stirring for about 5 min. The addition of 3 g of sodium nitrite (0.043 mol) was repeated twice more, keeping everything under magnetic stirrer for 1 h and 15 min. At the end of the third addition, 10 mL of formic acid was added to neutralize the sodium nitrite excess. The compound was then precipitated with 400 mL of cold ethyl ether and 100 mL of acetone under stirring for 30 min and was filtered and washed with distilled H_2_O and ethanol (100 mL). Subsequently, a further washing was carried out with diethyl ether and methanol to obtain a product which was dried under vacuum and characterized by FT-IR [39].

### 3.5. Determination of Carboxylic Groups Content

The carboxylate chitosan (0.05 g) was suspended in a solution consisting of 2.5 mL of phosphate buffer (pH 8.0) and 2.5 mL of aqueous methylene blue solution and then was filtered, acidified with 1 mL of HCl 0.1 N, and added with 8 mL of distilled water. The determination of the carboxylic groups was performed using a UV-Vis spectrophotometer through a method that involves the analysis of the test sample containing methylene blue. This sample bind carboxyl groups and tends to decrease its concentration within the solution; the resulting amount of unabsorbed methylene blue has been used in the following equation 1 to determine the actual content of carboxyl groups [40]:

(1) $$\mathrm{mmol}\;\mathrm{COOH}/g\;\mathrm{dry}\;\mathrm{sample}\;=\;\frac{\left(\;7.5\;-\;A\right)\;\times \;0.00313}{E}$$

A = non-absorbed amount of methylene blue;

E = CC amount in grams.

### 3.6. Synthesis of CSA-CC

In a two-necked flask under magnetic stirrer for about 1 h, 0.06 g (4.98 × 10^−5^ mol) of CsA, 1 mL of bromoacetyl bromide (0.013 mol) and 0.03 g of dimethylaminopyridine (2.45 × 10^−4^ mol) were added. The progression of the reaction was monitored by TLC (silica gel) using chloroform-methanol (6:4) as eluent mixture. At the same time, an aqueous solution containing 7.5 mL of distilled water and 1.15 g of NaHCO_3_ (0.0136 mol) was prepared. This last was added to the compound contained in the flask. The two phases were separated by performing an extraction with dichloromethane (6 mL). After that, the organic phase was treated with Na_2_SO_4_ to remove the residual water, washed with dichloromethane and then filtered. The product was evaporated, lyophilized, characterized to ^1^H-NMR to give 0.578 g. Successively, 0.578 g (4.4 × 10^−4^ mol) of product were added to 20 mL of DMF and 0.0310 g (4.78 × 10^−4^ mol) of sodium azide, to obtain a solution which was left under magnetic stirrer for 24 h. The mixture was evaporated, and the residue was dispersed in dichloromethane (60 mL) and washed with an aqueous solution containing NaCl. Then, the organic phase was a hydride with Na_2_SO_4_, filtered and evaporated. The residue was dried and dissolved in 2 mL of dry tetrahydrofuran under magnetic stirrer. In the mixture, 0.000143 g (5.45 × 10^−7^ mol) of tri-phenyl phosphine and 41 µL of distilled water were added and stirred for 18 h. The solvent was evaporated, and the obtained residue was dissolved in 100 mL of ether and in a cold solution of HCl in ether to facilitate its precipitation. The residue was dissolved in ethyl ether and n-hexane, evaporated, frozen and lyophilized. At the obtained product, 125 mL (1.95 mol) of dichloromethane, 0.37 mL (2.12 × 10^−3^ mol) of DIPEA, 0.044 g (2.83 × 10^−4^ mol) of EDC, 0.162 g (7.86 × 10^−4^ mol) and 0.172 g of CC were added. The reaction was kept under magnetic stirring for 24 h, then the product was filtered and dried under vacuum [41,42].

### 3.7. Preparation of Chitosan (CM) and Membranes Chitosan (CMCsCC) Membranes

Dense membranes consist of dense structure presenting no detectable pore at the limits of electron microscopy. A mixture of molecules is transported through dense membranes by diffusion under the driving force of a pressure, concentration, or electrical potential gradient. Dense membranes may have a symmetric or an asymmetric structure. Dense membranes were prepared by phase inversion induced by solvent evaporation. A mixture of 0.15 g of chitosan in 5 mL of acetic acid solution (3%) was stirred for 24 h in a vial placed inside an oil bath at 40 °C. One milliliter of this solution was then placed in a multi-well plates and dried at 80 °C for 4 h [43]. Table 1 shows other examples of membranes made with this procedure.

### 3.8. In Vitro Skin Permeation Studies

Skin permeation studies were performed by using Franz diffusion cells apparatus with cellulose acetate membranes and pig skin (furnished from local butcher) for 24 h. The apparatus was maintained at 37 °C to mimic physiological conditions. Receptor chambers (7.0 mL) were filled with NaCl 0.9% solution containing ethanol (20%) and kept under stirring. Unloaded chitosan membranes were used as control. At specific time intervals (1, 2, 4, 8, 10, and 24 h) an aliquot (1 mL) of each sample was withdrawn from receptor chambers and replaced with fresh release medium. Samples were analyzed through UV-Vis spectrophotometry and drug release profiles were expressed as percentage of drug released respective to the total loaded amount in function of time.

### 3.9. Localization of CsA in Skin (CLSM Study)

Confocal laser scanning microscopy (CLSM) (Leica Microsystems Srl, Milan, Italy) was carried out to see the depth of permeation of a fluorescent lipophilic substance as a model drug like coumarine-6. The dye loaded membranes were applied on the pig skin and kept for 24 h in the permeation experiment. At the end of the experiment, the excess formulation was removed from the skin surface. The skin was washed 3 times with phosphate buffer (pH 7.4) and dried. Specimens were embedded in optimal cutting temperature compound (Tissue-Tek, Sakura Finetek Europe, Alphen aan den Rijin, The Netherlands) and stored at −80 °C. Cryostat-cut skin sections (16 μm thick) were mounted on slides and nuclei counterstained with Vectashield solution containing 1.5 μg/mL 4′,6-diamidino2-phenylindole (DAPI; Vector Laboratories, Burlingame, CA, USA). Images were acquired using a confocal microscope (Leica TC-SP2 Confocal System, Leica Microsystem Srl, Milan, Italy).

### 3.10. Cell Proliferation Assays

MDA-MB-231 cells were seeded, in triplicate, in 6-well plates in a regular growth medium. On the second day, the cells were synchronized in serum free media (SFM) for 24 h, so that most of the cells belonged to a population in the same cell cycle phase, to avoid growth differences among cells. The following day, cells were put in contact with chitosan, CsA, carboxylated chitosan, CsACC and membrane based on chitosan + CsACC. After 72 h, MDA-MB-231 cells were harvested by trypsinization and collected in Eppendorf. All collected samples were incubated in a 0.5% trypan blue solution for 1 min at room temperature. Cell viability was determined by Countess Automated Cell Counter (NucleoCounter® NC-202, Gydevang, Denmark) [44].

## 4. Conclusions

The present work aimed to design and realize CsACC-based membranes that were potentially useful in the treatment of breast cancer in the form of subcutaneous implants. The membranes obtained were prepared starting from the polymeric product based on carboxylated chitosan and cyclosporin A. Such precursor was obtained by reaction of amidation of the active principle with the previous carboxylated chitosan and characterized by FT-IR and DSC. In vitro permeation studies showed that membranes could potentially release the cyclosporin-A in the skin internal layers. In addition, membranes containing both CsACC membranes and free CsA showed a significant decrease in human breast cancer MDA-MB-231 cell viability. This effect is not observed by treating the cells uniquely with free CsA at the same concentration with which it is present in the membranes. Therefore, the obtained membranes could be an interesting strategy for the delivery of cyclosporin-A in patients affected by breast cancer to limit its systemic toxicity.

## Figures and Tables

**Table 1:** Membranes based on chitosan and other components.

Membranes	Chitosan	Acetic Acid Solution (mL)	Csacc (g)
CM *^1^	0.15 g	5 mL	-
CM *^2^ + Csacc	0.15 g	5 mL	0.017 g

*^1^ Chitosan Membranes. *^2^ Chitosan Membranes + Ciclosporine A-Carboxylated Chitosan.

## Data Availability

Not applicable.

## Sample Availability

Samples of the compounds are available from the authors.
